# Mini-Implant-Retained Overdentures for the Rehabilitation of Completely Edentulous Maxillae: A Systematic Review and Meta-Analysis

**DOI:** 10.3390/ijerph18084377

**Published:** 2021-04-20

**Authors:** Serena Vi, Damon Pham, Yu Yian Marina Du, Himanshu Arora, Santosh Kumar Tadakamadla

**Affiliations:** 1 School of Medicine and Dentistry, Griffith University, Gold Coast 4222, Australia; serena.vi@griffithuni.edu.au (S.V.); damon.pham@griffithuni.edu.au (D.P.); marina.du@griffithuni.edu.au (Y.Y.M.D.); santoshkumar.tadakamadla@griffithuni.edu.au (S.K.T.); 2 School of Dentistry, The University of Queensland, Brisbane 4006, Australia; 3 Menzies Health Institute Queensland, Griffith University, Gold Coast 4222, Australia

**Keywords:** osseointegration, maxilla, survival rate, patient satisfaction

## Abstract

Purpose: Mini-dental implants (MDIs) have been used to support and retain overdentures, providing patients with a less invasive placement procedure. Although lucrative, the use of MDIs to retain a maxillary overdenture is still not an established treatment modality. This systematic review aims to answer the question: Do mini-implant-retained maxillary overdentures provide a satisfactory treatment outcome for complete edentulism? Methods: A systematic search for relevant articles was conducted to include articles published until April 2021 in the following electronic databases: CINAHL, Cochrane, EMBASE, PubMed, and Web of Science. All empirical studies evaluating the biological, survival, or patient-reported outcomes after placing mini-implant-retained overdentures in maxilla were considered for inclusion. The risk of bias was assessed by utilizing the Joanna Briggs Institute critical appraisal checklist. Study screening and data extraction were conducted by three reviewers independently. Results: The electronic search retrieved 1276 titles after omitting duplicates. Twenty articles were considered for full-text review, of which six studies were included in this systematic review. The included studies evaluated a total of 173 participants with a mean age of 66.3 years. The overall mini-implant survival rate was 77.1% (95% CI: 64.7–89.5%) with a mean follow-up time of 1.79 years (range: 6 months to 3 years). Implant survival differed significantly when comparing complete and partial palatal coverage overdentures. Those with complete palatal coverage exhibited less bone loss overall compared to partial coverage overdentures. Participants of all studies reported an increase in the quality of life and in satisfaction after rehabilitation treatment with MDIs. Conclusions: The survival rate of mini-implants retaining an overdenture in the maxilla was observed to be lower than the values reported for traditional implants in the literature. Improvements were observed in all aspects in terms of patient satisfaction, quality of life, oromyofunction, and articulation after the treatment.

## 1. Introduction

Edentulism, although not a disease process, is a reflection and, in some cases, the endpoint of past or current conditions such as periodontal disease, caries, or trauma. A study analyzing the global burden of oral conditions reported that, in 2017, the prevalence of global tooth loss in all age groups was 3.3% (267 million cases), with the highest prevalence rate in the geriatric population (85 to 89 years). In addition, the prevalence was lowest in low-income countries (8 million cases) and highest in upper-middle-income countries (120 million cases) [1]. Therefore, tooth loss is a common condition, with it being more prevalent, however not exclusive, amongst the geriatric population. Conventionally, the oral rehabilitation of completely edentulous patients has involved the use of complete dentures. Another treatment modality to replace missing teeth includes the use of dental implants, which are generally titanium surgical components that osseointegrate with the jawbones to support and retain a prosthesis [2]. In certain circumstances, dental implants can be utilized to retain a denture, also known as an implant-retained overdenture. Patients who have lost neuromuscular control to retain conventional dentures, or have difficulty in retaining conventional dentures due to xerostomia or parafunctional habits, may benefit from having implant-retained overdentures.

Implant overdentures offer various benefits that are not provided with conventional dentures. Patients with implant overdentures often report improved appearances, oral health-related quality of life (OHRQoL), and satisfaction [3]. Furthermore, implant overdentures offer increased biting forces over conventional dentures, enabling patients to eat a larger selection of foods, as well as preventing bone loss and muscle atrophy, common in edentulous patients [4]. However, due to the more costly and invasive nature of implant overdentures, medically compromised and geriatric patients may be precluded from this form of therapy. Standard implants are generally utilized for implant overdentures; though, more recently, the treatment modality of MDIs to support overdentures has become increasingly accepted [5,6]. MDIs have several distinguishable properties from standard implants; they are constructed with a reduced diameter (less than 3 mm); moreover, their design differs from the conventional two-piece implants in that they lack an abutment screw in favor of a single-piece design with a ball-shaped attachment to retain overdentures. MDIs are indicated when the use of standard implants is not possible for the retention of an overdenture, such as those with narrow alveolar ridges [7]. MDIs may also be considered for patients who require less invasive procedures, as they can be placed without the need for bone grafting, such as patients with narrow alveolar ridges where standard implants would need augmentation procedures [7]. Due to the less invasive nature of MDIs and the ability to place a reduced number of implants, their use has become a novel focus amongst implant research.

The rehabilitation of the maxilla is considerably different to that of the mandible due to the presence of thinner cortical bones and more trabecular bones. This poses certain challenges when it comes to osseointegration and survival and success rates of dental implants. In contrast to the mandible, implant overdentures in the maxilla have been less commonly studied and reported, resulting in contention and the lack of consensus regarding their effectiveness [8,9,10,11,12,13,14]. It is widely accepted that the use of four standard implants is the minimum to retain a maxillary overdenture [15,16,17,18,19]. Due to the limited studies in the literature reporting the use of MDIs to retain maxillary overdentures, this review aims to answer the research question: is the use of mini-implant-retained maxillary overdentures more effective in comparison to traditional implant-retained maxillary overdentures in the management of patients with complete edentulism? Specifically, this review will focus on biological outcomes, survival and success rates, patient satisfaction, and complications. 

## 2. Methods

This report follows the Preferred Reporting Items for Systematic Reviews and Meta-Analyses (PRISMA) guidelines. The protocol of this systematic review is registered with the International prospective register of systematic reviews under the registration ID CRD42020183009. This systematic review aims to compare mini-implant-retained maxillary overdentures to traditional implant-retained maxillary overdentures in the management of patients with maxillary edentulism. The population included in this review are participants with completely edentulous maxillae. The intervention group includes participants with mini-implant-retained overdentures. The outcomes assessed include biological outcomes, implant survival, prosthesis survival, patient satisfaction, and oral health-related quality of life. 

### 2.1. Data Sources

The search for relevant articles was conducted in April 2020 (updated in April 2021 in PubMed) in the following electronic databases: CINAHL, Cochrane, EMBASE, PubMed, and Web of Science. The search strategy used in PubMed is presented in Table 1, which was independently conducted by three reviewers (SV, DP, and MD). The search strategy was adapted according to the databases with no language or time filters. The applied inclusion and exclusion criteria are illustrated in Table 2.

### 2.2. Screening and Selection

All titles and abstracts were individually reviewed by three independent reviewers (SV, DP, and MD). Any discrepancies and disagreements were discussed and resolved by consensus from all three aforementioned independent reviewers. Selected articles were further screened for eligibility by full-text analysis independently by all three reviewers (SV, DP, and MD).

### 2.3. Data Extraction

Pre-piloted data extraction charts were used to extract the information from the selected studies, which included: study design, study setting, sample size, gender and age of the participants, patient inclusion and exclusion criteria, opposing dentition, implant system, MDI diameter, number of implants placed, type of attachment, denture design, surgical procedure, loading period, outcomes reported, data collection method, and number and length of follow-ups. The data extraction from the articles was conducted individually by two researchers independently (SV and DP). A third researcher (MD) reconfirmed the accuracy of the extracted data by cross-checking the information in the data extraction charts with the full-text articles.

### 2.4. Risk of Bias and Quality of Evidence

A Joanna Briggs Institute (JBI) critical appraisal checklist was deemed appropriate and was used to assess the risk of bias in the included studies [20]. It is used to evaluate the methodological quality of a study. It assesses the possible risk of bias in terms of design, conduct, and analysis by comprising 11 methodological items with each item rated with a yes, no, unclear, or not applicable [20]. A JBI critical appraisal checklist was conducted by two reviewers independently (DP and MD). A third reviewer (SV) was then included to resolve any disagreements and discrepancies by consensus.

### 2.5. Statistical Analysis

Survival rates were reported as a categorical variable. Raw data indicating the total number of implants survived in maxilla among the total number of implants placed at the end of the observation period (which ranged from 12 to 36 months) were extracted from five studies. A random-effects model was used to conduct meta-analysis by the arcsine transformation of the proportion of survival rate. Survival data at 12 months were available from four studies, and a meta-analysis including these four studies was also conducted to assess the survival rate at 12 months. The arcsine transformation is considered more appropriate than the logit transformation when the outcome is binary. The I^2^ statistic was used to assess the heterogeneity between the studies. OpenMetaAnalyst software was used to conduct the meta-analysis [21]. Publication bias was not assessed, as there were only a few studies in the meta-analysis.

## 3. Results

### 3.1. Literature Search

**Figure 1:**
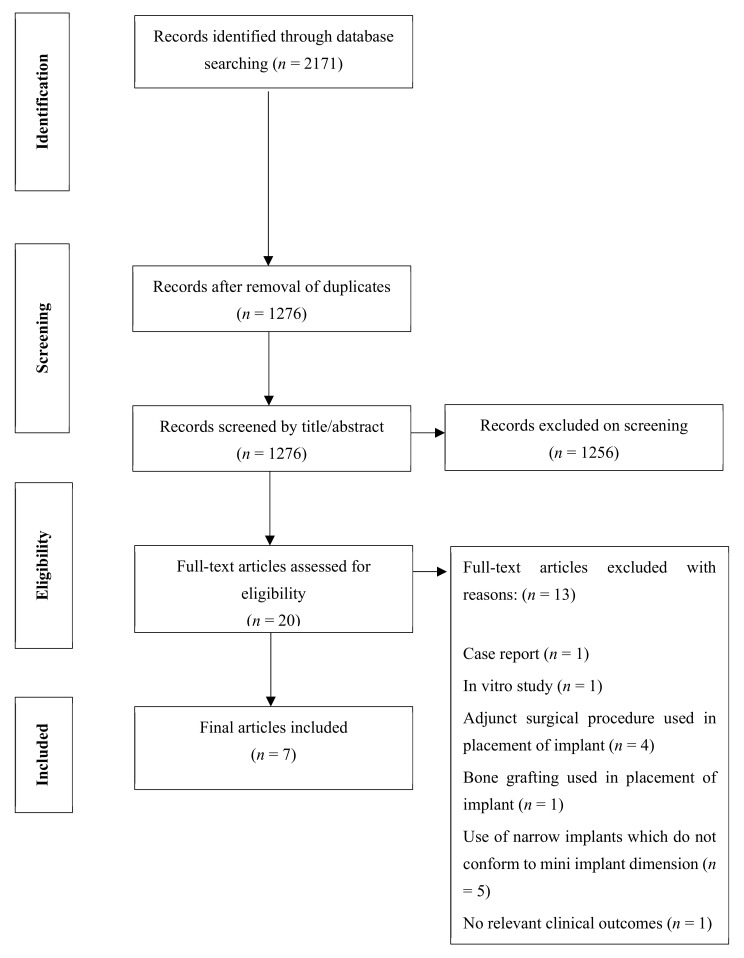
PRISMA flowchart.

The initial search performed in the selected databases yielded a total of 2171 articles, of which 1276 remained after omitting duplicates. An additional 1256 references were removed after a detailed review of the titles and abstracts, resulting in 20 full-text articles to be assessed for eligibility. With application of the eligibility criteria, a total of seven articles, six studies as two articles were of the same study, were selected for full-text review. A PRISMA flowchart depicting the flow of studies is displayed in Figure 1.

### 3.2. Study Characteristics

As depicted in Table 3, of the six included studies, one was retrospective, and the remaining five were prospective cohort studies. Overall, a total of 173 patients with a mean age of 66.3 years were included. In five studies, a total of 685 MDIs were placed in 148 participants, and the remaining one study [22] did not report the total number of implants. The mini-implant system varied across the seven studies; however, all utilised a flapless surgical procedure to place a one-piece ball type implant, and all but one study used an MDI diameter of 2.4 mm. A total of 6 MDIs in the maxilla for rehabilitation with an overdenture prosthesis was most commonly used across all studies. A complete denture was used in all cases, though both complete and partial coverage denture designs were used. Throughout all studies, the opposing dentition varied greatly. This included an opposing dentition with all partial or fully dentate mandibles with a combination of natural teeth and partial prostheses. One study included reports of both MDI overdenture treatments for the maxilla and mandible, while the remaining five studies exclusively focused on maxillary MDI overdentures.

### 3.3. Risk of Bias

As seen in Table 4, the majority of studies examined a single group of subjects with all subjects being exposed to the intervention; therefore, the first two questions from the JBI critical appraisal checklist, “Were the two groups similar and recruited from the same population?” and “Were the exposures measured similar to assign people to both exposed and unexposed groups?”, were nonapplicable and no biases could be ascribed. In contrast, many of the studies lost participants to follow-ups; however, two studies [23,24] did not describe reasons for the loss of follow-ups. Similarly, all studies did not ascribe any strategies to address incomplete follow-ups. From these findings, it is evident that the most notable form of bias detected was attrition bias. A loss of greater than 20% of participants was seen in two studies [22,25,26,27]. Of all the studies, which demonstrated incomplete follow-ups, no studies employed strategies to account for the loss in sample size. In contrast, all studies identified confounders, as well as employed an exclusion criterion to eliminate any confounders, such as those participants who possess medical comorbidities. Fonteyne et al. [22], as the exception, did not account for confounding factors. All studies utilized and applied appropriate statistical analyses. Furthermore, strategies were not employed to mitigate the effects of attrition bias. All studies, with the exception of Preoteasa et al. [23] and Elsyad et al. [13] (no loss to follow-up), had dropout rates greater than 5%. It has been argued that a follow-up loss of 5% or lower is of little significance [28].

**Figure 2:**
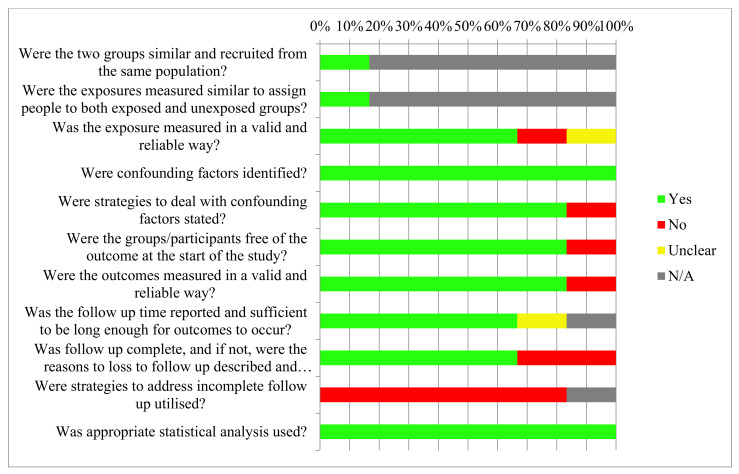
Risk of bias across studies.

Therefore, the most common bias observed among the included studies was attrition bias, as presented in Figure 2. In addition, the studies by Mundt et al. [25,26] were subject to biases, in which the subjects were not free from the outcome at the start of the study; however, this is a flaw resulting from the type of study conducted.

### 3.4. Effect of MDIs on Biological Outcomes

The most common biological outcome investigated was changes in marginal bone levels [13,23,25,26]. As seen in Table 5, in the study by Mundt et al. [25], a total of 13.6% of MDIs encountered bone loss greater than 2 mm where, more specifically, 3.3% experienced a total bone loss of 3 mm or greater. Meanwhile, in the study conducted by Preoteasa et al. [23], where patients were treated with complete palatal coverage dentures, bone loss was measured in threads, with 11 MDIs exhibiting bone loss of one or more threads. When looking at the effect of the amount of palatal coverage on bone level changes, one study reported that patients with complete palatal coverage overdentures showed less bone loss overall, when compared to partial palatal coverage, although it was not statistically significant [13].

### 3.5. Effect of MDIs on Implant Survival

Five of the six studies evaluated the implant survival or success or failure rates as depicted in Table 5 [13,24,25,26,29]. The overall range of survival rates among these studies recorded 53.8% as lowest and 94.3% as highest. At the end of each of the five studies’ follow-up periods, the reported survival rates were Elsyad et al. [13] (Group I and II, 78.4% and 53.8%, respectively), Van Doorne et al. [24] (82.3%), Mundt et al. [25,26] (93.4%), Tomasi et al. [29] (57.1%), and Preoteasea et al. [23] (77.8%). In the study carried out by Elsyad et al. [13], MDI survival rates were higher for full palatal coverage (78.4%) when compared to partial palatal coverage (53.8%). However, when comparing the results of Preoteasa et al. [23] and Van Doorne et al. [24], where they exclusively reviewed complete and partial palatal coverage, respectively, the survival rate for partial palatal coverage (82.3%) was higher than that for full palatal coverage (77.8%). Articles investigating both maxillary and mandibular arches showed MDI failure rates significantly higher in the maxillary arch when compared to the mandibular arch [24,25,26,29]. The greatest MDI failure rate was observed during the initial insertion healing phase [13,24,26].

**Figure 3:**
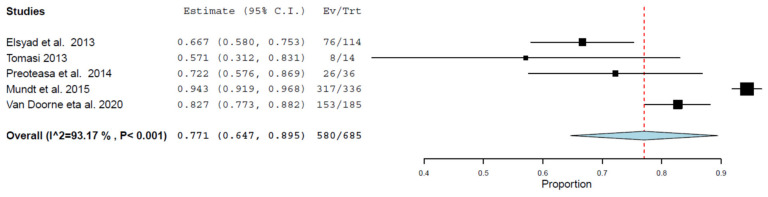
Meta-analysis for MDI survival based on data reported at the final follow-up.

A meta-analysis was conducted on the survival rates. Figure 3 demonstrates an overall survival rate of 77.1% (95% confidence intervals: 64.7–89.5%) based on the data from the included studies on MDIs in the maxillary arch that had a follow-up range of 12–36 months; the effect size was significant (*p* < 0.001). There was a significant statistical heterogeneity between the studies (I^2^ = 93.17%, *p* < 0.001).

### 3.6. Effect of MDIs on Prosthesis Survival

The overdenture status was recorded in only two studies [23,25]. Of these two studies, only one out of the 78 included overdentures fractured across both studies, while the remaining 77 overdentures were consistently functional over the observation period [23,25]. The fracture recorded occurred in the area between the locations of MDIs [23].

### 3.7. Effect of MDIs on Patient Satisfaction and OHRQoL

The degree of patient satisfaction or OHRQoL was mostly evaluated after patients received rehabilitation treatment with the MDI-supported overdenture. Patient satisfaction was assessed using the Visual Analog Scale (VAS), which was the most frequently applied index [13,22,29], while closed-ended questions were used in two studies [24,29]. OHRQoL was also evaluated in two studies, which employed the Oral Health Impact profile (OHIP-14) [22,25,26].

The definitive overdenture prosthesis resulted in an increase in satisfaction across many oral health-related factors, including general speech and articulation profiles [22,23,29], oromyofunction and chewing ability [13,23,29], retention [13,23,25,29], and aesthetics [22]. A rise in patient satisfaction with treatment was also observed at the final prosthesis connection and the termination of treatment across four studies [22,24,25,26,29]. When comparing full and partial coverage overdentures, full palatal coverage was reported to provide superior retention; however, no significant difference in mastication was recorded [13].

### 3.8. Association of Confounding Variables and MDI Success

The association of confounding variables on the overall MDI and overdenture status was mentioned across four included studies [23,24,25,26]. Two studies reported a relationship between patient, gender, and one of the outcome variables. In the first study, one MDI loss was higher in females (26.5%) when compared to males (9.8%) [24], while marginal bone loss was reported to be more severe in women in the second study [23]. The marginal bone loss around implants was recorded to be higher in patients who were former smokers, those with decreased bone densities, and those with decreased ridge widths [23,25,26]. Moreover, the effect of insertion torque on the MDI survival rate and bone loss was reported by two studies [23,24]. Insertion torques between 15 and 25 N recorded the highest survival rates [24], and implants inserted with lower torque values experienced increased marginal bone losses [23].

## 4. Discussion

This systematic review evaluated the available evidence on the use of MDI as a suitable alternative treatment modality to traditional implants for maxillary overdentures. MDIs are predominately used for medically compromised patients, who may not be suitable candidates for surgical procedures and, thus, traditional implant placement [30]. The application of MDIs in the mandibular alveolar ridge provides an improved stability and retention of overdentures; however, there are limited studies in regard to the use of MDIs in the retention of overdentures in the maxillary arch [30]. To the best of our knowledge, this is the first review that attempts to synthesize the available evidence to assess the effectiveness of MDI-retained overdentures in the maxilla.

The survival rate for MDI in the maxilla was found to be 77.1% from our meta-analysis, which is lower than the 98.1% survival rate with traditional implants, as reported in the systematic review conducted by Raghoebar et al. [31]. The loss of MDI can be attributed to poor primary stability during osseointegration and excessive load, resulting in fatigue fracture [32]. A study conducted by Allum et al. [32] displayed an increased risk of fracture in MDI compared to regular diameter implants. A wide range of survival rates was observed among the five studies that reported survival rates in the current review. The difference may be attributed to the number of implants placed per arch. In a systematic review aiming to explore the number of implants required for maxillary overdentures, Di Francesco et al. [33] concluded that the survival rate was high when four or more implants were placed, while the number of implants was not related to patient satisfaction. An increase in the number of implants allows for increased load distributions and, hence, decreased biomechanical forces per implant [34]. In the studies conducted by Preoteasa et al. [23] and Van Doorne et al. [24], an opposing dentition with the combination of natural teeth and partial prosthesis demonstrated the highest MDI survival rate, and the lowest survival rates being associated with implant-supported overdenture antagonists. This may be contributed with the difficulties in achieving bilateral balanced occlusion with two occluding complete dentures [35]. Occlusal discrepancies during function may result in rapid bone loss and failure of the MDIs [35].

When assessing the amount of palatal coverage for MDI-retained maxillary overdentures, a comparative study was conducted by Elsyad et al. [13] comparing partial versus full palatal coverage designed dentures. Complete palatal coverage presented with a higher implant survival rate as opposed to partial palatal coverage. The addition of palatal coverage provides supplementary support of the overdenture and improves the biomechanical distribution between implants and adjacent soft and hard tissues [36]. Considering no implants were splinted, an increased amount of forces falling on individual fixtures is a possibility. Consequently, the changes in bone levels recorded in this study support the above reasoning. The resultant mean bone loss was higher in palatal-less coverage designs than full-palatal coverage. When looking at prosthesis survival, all but one fabricated denture remained in function throughout the observation period. A fractured denture was reported by Preoteasa et al. [23], where the prosthesis fractured in an area located between the implants of a complete palatal coverage denture. Partial palatal coverage dentures, also known as horseshoe dentures, may include a metal reinforcement, which reduces the risk of fracture and deformation of the denture [34].

Overall, across all six included studies, patients were generally satisfied with their denture when reviewing aesthetics, retention, and function. The satisfaction can be attributed to the improvement in mastication and chewing efficiency and, consequently, improvements were observed pre- and post-treatment for patient satisfaction and OHRQoL. In terms of oromyofunctional and articulation pre-treatment and post-treatment, patients exhibited a decrease in speech problems, with the majority of participants reporting little to no impact of speech problems with their overdentures. This finding seems prudent as edentulism is associated with the impairment of speech. The positioning of the teeth and the slurring action of the tongue against the teeth and palate are required for proper articulation [37].

The limitations of this systematic review pertain to the presence of bias, the lack of study homogeneity, the limited number of studies observed, and, to a smaller extent, the short follow-up length. In particular, attrition bias was one of the most notable forms of bias detected. The data collection method significantly varied between the studies; for example, patient satisfaction was measured using the VAS scale by some studies, while a dichotomous response was used in another. Moreover, varying outcomes, implant systems, denture designs, loading periods, and follow-up lengths also contributed to the heterogeneous nature of the studies examined in this review. Although the length of the follow-up period amongst the included studies only ranged between 6 and 36 months, this period is of high significance as it represents the period of the early phase of osseointegration that is associated with a higher risk of implant failure [38]. Beyond this period, many implants are lost as a result of peri-implant diseases, which cannot be observed with the shorter follow-up periods [39].

## 5. Conclusions

The survival rate of mini-implants retaining an overdenture in the maxilla was observed to be lower than the values reported for traditional implants in the literature. However, improvements were observed in all aspects in terms of patient satisfaction, quality of life, oromyofunction, and articulation after the treatment with MDI. The few studies with shorter follow-up periods necessitate further studies with longer study periods and larger sample sizes.

## Figures and Tables

**Table 1:** Search strategy in PubMed.

#	Search Term
**#1**	Maxilla [MeSH term] OR Maxilla [All Fields]
**#2**	Maxillary [All Fields]
**#3**	(#1 OR #2)
**#4**	Mini dental implant* [All Fields]
**#5**	Narrow dental implant * [All Fields]
**#6**	Mini implant* [All Fields]
**#7**	Narrow implant* [All Fields]
**#8**	Mini implant overdenture* [All Fields]
**#9**	Narrow implant overdenture* [All Fields]
**#10**	Mini implant supported overdenture* [All Fields]
**#11**	Narrow implant supported overdenture* [All Fields]
**#12**	Mini implant supported denture* [All Fields]
**#13**	Narrow implant supported denture [All Fields]
**#14**	Mini implant overlay denture* [All Fields]
**#15**	Narrow implant overlay denture* [All Fields]
**#16**	Mini implant supported overlay denture* [All Fields]
**#17**	Narrow implant supported overlay denture* [All Fields]
**#18**	Full mouth rehabilitation [All Fields]
**#19**	(#4 OR #5 OR #6 OR #7 OR #8 OR #9 OR #10 OR #11 OR #12 OR #13 OR #14 OR #15 OR #16 OR #17 OR #18)
**Final Search**	#3 AND #19

**Table 2:** Inclusion and exclusion criteria.

Selection Criteria	Inclusion Criteria	Exclusion Criteria
**Language type**	English	Non-English
**Study type**	Empirical studies including randomised controlled trials, nonrandomised clinical trials, cohort studies, case series	Review studies including narrative reviews, systematic reviews, literature reviews
**Study Features**	Flapless placement of implants Implant diameter up to and including 2.4 mm Mini implant-retained overdentures Maxillary cases Partial and complete edentulism	Adjunct surgical procedures used for implant placement Implant diameter of 2.4 mm and more Overdentures retained via other means Fixed partial dentures Mandibular cases
**Analysis of clinical outcomes**	At least one of the following parameters: Patient satisfaction and perception in regard to treatment Success and survival rate	Studies focusing on participants pre-treatment characteristics
**Publication date**	Publications from 2010 to present	Publications prior to 2010
**Population**	Human studies (all ages)	In vitro studies Animal studies
**Selection criteria**	Inclusion Criteria	Exclusion Criteria

**Table 3:** Study design of the included studies.

Author (Year)	Design	Study Setting	Sample Size and Dropout Rates	Mean Age and Gender (M:F)	Opposing Dentition	Implant System	Number of Implants Placed Per Patient in Maxilla	Denture Design	Loading Period	Number of Follow-Ups	Length of Follow-Up
Fonteyne (2019)	Prospective cohort study	University Clinic Ghent and General Hospital AZ ZENO Knokke-Blankenberge, Belgium	25; 21.9%	62.6; 17:13	Natural teeth and combination of natural teeth and partial prosthesis	ILZ Southern Implant Inc.	5 to 6	Complete denture with partial palatal coverage	6 months	Two: At provisional loading and final connection	6 months
Elsyad (2013)	Prospective cohort study	Mansoura University, Mansoura, Egypt	19; 0.0%	63.8; 11:8	All patients were given new mandibular dentures	MAX Thread, Sendaxs MDI, IMTECT	6	Group I—Full palatal coverage Group II—Partial palatal coverage	Immediate- same day	Three: 6, 12, and 24 months after insertion	24 months
Van Doorne (2020)	Prospective cohort study	University Clinic Ghent and General Hospital AZ ZENO Knokke-Blankenberge, Belgium	31; 6.5%	62.3; 17:14	Natural teeth and combination of natural teeth and partial prosthesis	ILZ Southern Implants Inc.	6	Complete denture with horseshoe design	6 months	Six: 1 week, followed by 1, 3, 6, 12, and 24 months	24 months
Mundt (2013, 2015)	Retrospective study	Nine private practices in Germany	54; 26.9%	71.2; 54:79	Not specified	3M ESPE	4, 5, 6, 7, 8, or 10	Complete dentures with complete and partial palatal coverage	Immediate or delayed with 3 to 4 months	Not specified	27.1 ± 12.8 months
Preoteasa (2014)	Prospective cohort study	University of Medicine and Pharmacy, Bucharest, Romania	23; 4.2%	62.0; 10:13	Natural teeth or fixed prosthetic restoration	IMTEC/3M ESPE	5 or 6	Complete denture with complete palatal coverage	Not specified	Six: Weekly during 1st month post-surgery,3 and 6 months, and 1, 2, and 3 years post-surgery	36 months
Tomasi (2013)	Prospective cohort study	University of Gothenburg, Gothenburg and private practice, Mjolby, Sweden	21; 8.7%	71.0; 9:12	Not specified	Dentatus Atlas	2 and 4	All complete dentures with full palatal coverage	Not specified	One: 12 months post-treatment	12 months

**Table 4:** Risk of bias within studies.

	Were the Two Groups Similar and Recruited from the Same Population?	Were the Exposures Measured Similar to Assign People to both Exposed and Unexposed Groups?	Was the Exposure Measured in a Valid and Reliable Way?	Were Confounding Factors Identified?	Were Strategies to Deal with Confounding Factors Stated?	Were the Groups/Participants Free of the Outcome at the Start of the Study?	Were the Outcomes Measured in a Valid and Reliable Way?	Was the Follow-Up Time Reported and Sufficient to be Long Enough for Outcomes to Occur?	Was the Follow-up Complete, and If Not, Were the Reasons for Loss to Follow-Up Described and Explored?	Were Strategies to Address Incomplete Follow-Up Utilised?	Was Appropriate Statistical Analysis Used?
Fonteyne, E., et al. (2019)	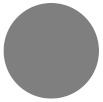	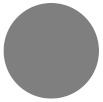	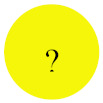		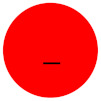			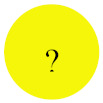		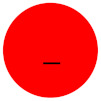	
Elsyad, L., et al. (2013)										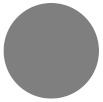	
Mundt, T., et al. (2013 and 2015)	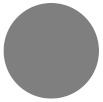	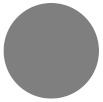				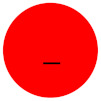		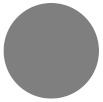		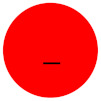	
Preoteasa, E., et al. (2014)	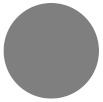	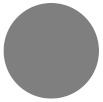							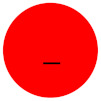	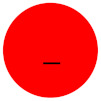	
Tomasi, C., et al. (2013)	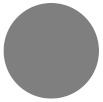	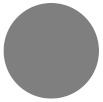	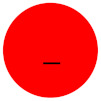							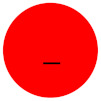	
Van Doorne, L., et al. (2020)	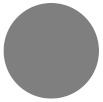	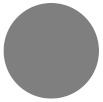					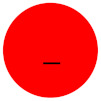		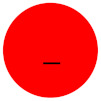	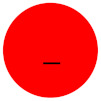	

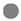
—Not applicable; 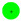
—Yes; 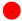
—No; 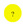
—Unclear.

**Table 5:** Study results of the included studies.

Author (Year)	Outcomes Reported	Data Collection Method	Length of Follow-Up	Biological Outcomes	Implant Survival and Success Rates	Patient Satisfaction and Oral Health-Related Quality of Life (OHRQoL)
Fonteyne (2019)	-Articulation - Oromyofunctional behaviour - Patient satisfaction - Quality of life	**Articulation:** Digitally with video camera **Oromyofunction:** 3-point rating scale **Patient satisfaction:** with oral health and speech via 10 cm VAS scale **OHRQoL:** OHIP-14	6 months	Not specified.	Not specified.	Satisfaction with oral health: increased from 67% to 83% Satisfaction with speech: increased from 77% to 84% OHRQoL: improved- OHIP score decreased from 21.97 to 8.23.
Elsyad (2013)	- Peri-implant bone loss around MI - Mobility - Patient satisfaction - Survival rate of MI	**Peri-implant bone loss:** Radiographically **Mobility:** Periotest instrument **Patient satisfaction:** with retention and chewing measured via 10 cm VAS scale **Survival rate:** Kaplan–Meier life table analysis	24 months	Mean vertical bone loss: Group I- 5.38 ± 1.65 mm Group II- 6.29 ± 2.33 mm Mobility: increased over time in both groups. Mobility of Group II > Group I at T2 and T3	Survival rate: Group I—78.4% Group II—53.8% (significant difference)	Patient satisfaction with retention: Group I-2.5 ± 0.84; Group II—1.0 ± 0.99 (no significant difference) Patient satisfaction with chewing: Group I- 7.9 ± 0.73; Group II- 8.22 ± 0.97 (significant improvement in both groups).
Van Doorne (2020)	- Implant stability at surgery - Patient’s perception of pain - Patient satisfaction - Survival rate - Success rate	**Implant stability:** Torque wrench **Perception of pain:** Numeric rating scale of 1 to 10 **Patient satisfaction:** ‘yes’/ ‘no’ questionnaire **Survival rate:** Kaplan–Meier life table analysis **Success rate:** When >5 MDIs are lost.	24 months	Not specified.	Survival rate: 82.3% Success rate: 93.5% Initial MDI failure rate: 17.3%. All MDI failures occurred in initial healing phase.	Average final patient satisfaction score was 8.6 ± 1.7. 96% of the patients would recommend this treatment to others.
Mundt (2013, 2015)	- Surgical and prosthetic complications - OHRQoL- Implant and prosthetic status Changes in bone level	**Surgical and prosthetic complications:** Data from patient records, oral examinations, and questionnaires. **OHRQoL:** OHIP-14 scale **Implant and prosthetic status:** Compared with participants records **Changes in bone level:** Radiographically	27.1 ± 12.8 months 2.2 ± 1 years	10.3% MDIs TBL of >2 mm 3.3% MDIs TBL of >3 mm	Survival rate: 94.3% 15 maxillary implants lost after insertion.	Significant improvements for all participants in all single questions regarding OHRQoL Retention rated very high in 9 (16.7%), fair in 44 (81.5%), and low in one denture.
Preoteasa (2014)	-MDI status - Overdenture status - Patient’s perception regarding treatment	**MDI Status:** Radiographically, clinical evaluation, self-reported peri-implant bleeding. **Overdenture status:** Self-reported **Patient perception:** Self-reported	36 months	11 MDIs presented with bone loss >1 thread.	MDI health status: 8 failed	Patients generally satisfied with aesthetics, retention, function (mastication and phonation). Complaints mainly associated with occasional pain to soft tissue supporting overdenture.
Tomasi (2013)	- Patient satisfaction - Clinical assessments - Survival rate	**Patient satisfaction:** 10 cm VAS scale and ‘yes’/‘no’ questionnaire **Clinical assessment:** Plaque score, BoP, and implant stability judged by percussion	12 months	Mean plaque score of 20% for maxillary and mandibular MDIs. Mean BoP score of 30%. Mean pocket probing depth was 2.3 mm with a range from 1 to 6 mm.	Implant failure rate was significantly higher in maxilla than the mandible, 43% and 15%, respectively. 6 out of 14 implants failed	All patients said ‘yes’ when asked if they were satisfied with their denture. Patient satisfaction with chewing ability increased from 4.5 to 9.0 Patient satisfaction with speech increased from 5.8 to 9.3 Patient overall denture satisfaction increased from 3.3 to 9.0

## References

[1] Bernabé E., Marcenes W., Hernandez C.R., Bailey J., Abreu L.G., Alipour V., Amini S., Arabloo J., Arefi Z., Arora A. (2020). Global, Regional, and National Levels and Trends in Burden of Oral Conditions from 1990 to 2017: A Systematic Analysis for the Global Burden of Disease 2017 Study. J. Dent. Res..

[2] Misch C. (2005). Dental Implants Prosthetics.

[3] Laurito D., Lamazza L., Spink M.J., De Biase A. (2012). Tissue-supported dental implant prosthesis (overdenture): The search for the ideal protocol. A literature review. Ann. Stomatol..

[4] Goodacre C.J. (2018). Implant overdentures: Their benefits for patients. Saudi J. Med. Med. Sci..

[5] Sivaramakrishnan G., Sridharan K. (2017). Comparison of patient satisfaction with mini-implant versus standard diameter implant overdentures: A systematic review and meta-analysis of randomized controlled trials. Int. J. Implants Dent..

[6] Lemos C.A.A., Verri F.R., Batista V.E.D.S., Júnior J.F.S., Mello C.C., Pellizzer E.P. (2017). Complete overdentures retained by mini implants: A systematic review. J. Dent..

[7] Upendran A., Gupta N., Salisbury H. (2019). Dental, Mini-Implants.

[8] Šćepanović M., Calvo-Guirado J.L., Marković A., Delgado-Ruiz R., Todorović A., Miličić B., Mišić A.T. (2012). A 1-year prospective cohort study on mandibular overdentures retained by mini dental implants. Eur. J. Oral Implantol..

[9] Abou-Ayash S., Enkling N., Srinivasan M., Haueter M., Worni A., Schimmel M. (2019). Evolution of in vivo assessed retention forces in one-piece mini dental implant-retained mandibular overdentures: 5-Year follow-up of a prospective clinical trial. Clin. Implants Dent. Relat. Res..

[10] Ahn M.-R., An K.-M., Choi J.-H., Sohn D.-S. (2004). Immediate Loading with Mini Dental Implants in the Fully Edentulous Mandible. Implant. Dent..

[11] Singh R.D., Ramashanker P.C. (2010). Management of atrophic mandibular ridge with mini dental implant system. Natl. J. Maxillofac. Surg..

[12] Aunmeungtong W., Kumchai T., Strietzel F.P., Reichart P.A., Khongkhunthian P. (2017). Comparative Clinical Study of Conventional Dental Implants and Mini Dental Implants for Mandibular Overdentures: A Randomized Clinical Trial. Clin. Implant. Dent. Relat. Res..

[13] Elsyad M.A., Ghoneem N.E., El-Sharkawy H. (2013). Marginal bone loss around unsplinted mini-implants supporting maxillary overdentures: A preliminary comparative study between partial and full palatal coverage. Quintessence Int..

[14] Kabbua P., Aunmeungtong W., Khongkhunthian P. (2020). Computerised occlusal analysis of mini-dental implant-retained mandibular overdentures: A 1-year prospective clinical study. J. Oral Rehabil..

[15] Slot W., Raghoebar G.M., Vissink A., Slater J.J.H., Meijer H.J. (2010). A systematic review of implant-supported maxillary overdentures after a mean observation period of at least 1 year. J. Clin. Periodontol..

[16] Sadowsky S.J. (2007). Treatment considerations for maxillary implant overdentures: A systematic review. J. Prosthet. Dent..

[17] Mericske-Stern R. (1998). Treatment outcomes with implant-supported overdentures: Clinical considerations. J. Prosthet. Dent..

[18] Mericske-Stern R.D., Taylor T.D., Belser U. (2000). Management of the edentulous patient. Clin. Oral Implant. Res..

[19] Kiener P., Oetterli M., Mericske E., Mericske-Stern R. (2002). Effectiveness of maxillary overdentures supported by implants: Maintenance and prosthetic complications. Int. J. Prosthodont..

[20] Moola S., Munn Z., Tufanaru C., Aromataris E., Sears K., Sfetcu R., Aromataris E., Munn Z. (2017). Chapter 7: Systematic Reviews of Etiology and Risk. Joanna Briggs Institute Reviewer’s Manual.

[21] Wallace B.C., Dahabreh I.J., Trikalinos T.A., Lau J., Trow P., Schmid C.H. (2012). Closing the Gap between Methodologists and End-Users: R as a Com-putational Back-End. J. Stat. Softw..

[22] Fonteyne E., Van Doorne L., Becue L., Matthys C., Bronckhorst E., De Bruyn H. (2019). Speech evaluation during maxillary mini-dental implant overdenture treatment: A prospective study. J. Oral Rehabil..

[23] Preoteasa E., Imre M., Preoteasa C. (2014). A 3-Year Follow-up Study of Overdentures Retained by Mini–Dental Implants. Int. J. Oral Maxillofac. Implant.

[24] Van Doorne L., De Kock L., De Moor A., Shtino R., Bronkhorst E., Meijer G., De Bruyn H. (2020). Flaplessly placed 2.4-mm mini-implants for maxillary overdentures: A prospective multicentre clinical cohort study. Int. J. Oral Maxillofac. Surg..

[25] Mundt T., Schwahn C., Biffar R., Heinemann F. (2015). Changes in Bone Levels around Mini-Implants in Edentulous Arches. Int. J. Oral Maxillofac. Implant..

[26] Mundt T., Schwahn C., Stark T., Biffar R. (2015). Clinical response of edentulous people treated with mini dental implants in nine dental practices. Gerodontology.

[27] Fergusson D., Aaron S.D., Guyatt G., Hébert P. (2002). Post-randomisation exclusions: The intention to treat principle and exclud-ing patients from analysis. BMJ.

[28] Kristman V., Manno M., Côté P. (2003). Loss to Follow-Up in Cohort Studies: How Much is Too Much?. Eur. J. Epidemiol..

[29] Tomasi C., Idmyr B.-O., Wennström J.L. (2013). Patient satisfaction with mini-implant stabilised full dentures. A 1-year prospective study. J. Oral Rehabil..

[30] Bidra A.S., Almas K. (2013). Mini implants for definitive prosthodontic treatment: A systematic review. J. Prosthet. Dent..

[31] Raghoebar G.M., Meijer H.J., Slot W., Slater J.J., Vissink A. (2014). A systematic review of implant-supported overdentures in the edentulous maxilla, compared to the mandible: How many implants?. Eur. J. Oral Implantol..

[32] Allum S.R., Tomlinson R.A., Joshi R. (2008). The impact of loads on standard diameter, small diameter and mini implants: A comparative laboratory study. Clin. Oral Implant. Res..

[33] Di Francesco F., De Marco G., Carnevale U.A.G., Lanza M., Lanza A. (2019). The number of implants required to support a maxillary overdenture: A systematic review and meta-analysis. J. Prosthodont. Res..

[34] Takahashi T., Gonda T., Maeda Y. (2016). Effects of Reinforcement on Denture Strain in Maxillary Implant Overdentures: An In Vitro Study under Various Implant Configurations. Int. J. Oral Maxillofac. Implant..

[35] Pommer B., Krainhöfner M., Watzek G., Tepper G., Dintsios C.-M. (2012). Relevance of Variations in the Opposing Dentition for the Functionality of Fixed and Removable Partial Dentures: A Systematic Review. Int. J. Dent..

[36] Ando T., Maeda Y., Wada M., Gonda T. (2014). Contribution of the palate to denture base support: An in vivo study. Int. J. Prosthodont..

[37] Molly L., Nackaerts O., Vandewiele K., Manders E., Van Steenberghe D., Jacobs R. (2007). Speech adaptation after treatment of full edentulism through immediate-loaded implant protocols. Clin. Oral Implant. Res..

[38] Pommer B., Frantal S., Willer J., Posch M., Watzek G., Tepper G. (2011). Impact of dental implant length on early failure rates: A meta-analysis of observational studies. J. Clin. Periodontol..

[39] Manor Y., Oubaid S., Mardinger O., Chaushu G., Nissan J. (2009). Characteristics of Early Versus Late Implant Failure: A Retrospective Study. J. Oral Maxillofac. Surg..

